# Histopathological and biochemical evaluation of the protective efficacy of *Prunus spinosa* L. extract in a rat model of indomethacin-induced gastric ulcer

**DOI:** 10.22038/ijbms.2024.78382.16941

**Published:** 2024

**Authors:** Nihal Cetin, Esma Menevse, Cengizhan Ceylan, Zeliha Esin Celik, Neriman Akdam, Seyma Tetik Rama, Tugsen Buyukyildirim, Leyla Pasayeva, Osman Tugay, Meltem Gumus

**Affiliations:** 1 Department of Pharmacology, Faculty of Medicine, Selcuk University, 42131, Konya, Turkey; 2 Department of Biochemistry, Faculty of Medicine, Selcuk University, 42131, Konya, Turkey; 3 Department of Clinical Pharmacy, Faculty of Pharmacy, Selcuk University, 42100, Konya, Turkey; 4 Department of Pathology, Faculty of Medicine, Selcuk University, 42131, Konya, Turkey; 5 Department of Biostatistics, Faculty of Medicine, Selcuk University, 42131, Konya, Turkey; 6 Research Assistant, Department of Pharmacology, Faculty of Pharmacy, Selcuk University, 42100, Konya, Turkey; 7 Research Assistant, Department of Pharmacognosy, Faculty of Pharmacy, Selcuk University, 42100, Konya, Turkey; 8 Department of Pharmacognosy, Faculty of Pharmacy, Erciyes University, 38280, Kayseri, Turkey; 9 Department of Pharmaceutical Botany, Faculty of Pharmacy, Selcuk University, 42100, Konya, Turkey; 10 Department of Pediatrics, Division of Pediatric Gastroenterology, Faculty of Medicine, Selcuk University, 42131, Konya, Turkey

**Keywords:** Gastric ulcer, LC-HRMS, Prunus spinose, PGE2, TNF-α

## Abstract

**Objective(s):**

Some species of *Prunus* L. are popularly used to treat gastric ulcers. However, the possible healing mechanisms of the anti-ulcer activity of *P. spinosa*, which has proven antioxidant, anti-inflammatory, and wound-healing properties, are unclear.

**Materials and Methods:**

Ethanol extracts of *P. spinosa* fruits were administered orally at 100 mg/kg and 200 mg/kg to Wistar albino rats, with an indomethacin-induced gastric ulcer model. The ulcerous areas on the stomach surface were examined macroscopically. Tissues were examined histopathologically and biochemically. LC-HRMS revealed the phytochemical content.

**Results:**

TNF-α, IL-6, IL-1β, IL-8, and NF-kB levels were higher in the gastric ulcer group than in the extract groups. The VEGF values did not differ in each group. A significant difference was found between the lansoprazole group and the high-dose *P. spinosa* group regarding PGE2 levels. A histopathologically significant difference was observed between the healthy group and the indomethacin-applied groups in terms of neutrophilic infiltration of the gastric mucosa. Ascorbic acid (1547.521 µg/g), homoprotocatechuic acid (1268.217 µg/g), and genistein (1014.462 µg/g) were found as the main compounds in the *P. spinosa* extract by LC-HRMS.

**Conclusion:**

Our results demonstrated that *P. spinosa* protected the gastric mucosa from inflammation and also modulated the PGE2 pathway. When considered in terms of TNF-α, IL-1β, IL-8, IL-6, PGE2, and NF-kB values, it can be concluded that it has a similar or even more positive effect than the reference substance. *P. spinosa* showed its effects in a dose-dependent manner.

## Introduction

Peptic ulcer disease (PUD) is a global public health issue characterized by inflammatory processes and ulcer formation, causing damage to the defensive barrier in the epithelial mucosa of the stomach and duodenum (1). The incidence of PUD is usually higher in people aged 30-60 years (2). It is known that PUD affects approximately 5-10% of the world’s population at varying rates depending on age, gender, existing diseases, drugs used, and geographical location. Multifactorial risks such as *Helicobacter pylori* infection, nonsteroidal anti-inflammatory drugs (NSAIDs) use, stress and lifestyle, smoking, caffeine, and alcohol consumption constitute the etiology of PUD (3, 4). Cytoprotective prostaglandins, bicarbonate, nitric oxide, etc., are used as protective factors for the stomach in PUD (4) The most common symptoms of PUD, which is an important cause of morbidity and mortality, are epigastric pain, dyspepsia, nausea, and bloating (5). Serious complications such as perforation, gastrointestinal bleeding, gastrointestinal obstruction, and malignancy may occur with PUD, and the ulcer treatment process must be successfully managed to prevent complications (6). Studies show that NSAID use may be responsible for 50% of PUD formation (7). One of the main risk factors in ulcer formation is using NSAIDs. It has been stated that NSAID group drugs give rise to the growth of gastric mucosal lesions by producing and depleting reactive oxygen species (ROS) of endogenous prostaglandins through inhibition of the cyclooxygenase (COX) enzyme. Thus, the cyclooxygenase enzymes and the synthesis of endogenous prostaglandins are inhibited by NSAIDs. Therefore, mucosal blood flow, mucosal resistance, and epithelial proliferation are decreased, resulting in gastric damage (8). 

Proton pump inhibitors (PPIs), prostaglandin analogs (misoprostol), and histamine-2 receptor antagonists (H2RAs) are frequently used in the clinic for the treatment of PUD and the prevention of its complications (9). Side effects due to long-term use of PPIs include susceptibility to infections, hypergastrinemia, chronic kidney and liver diseases, dementia, and bone fractures(10-12). It is also known to cause malabsorption by affecting the absorption of calcium, vitamin B12, iron, and magnesium (11). It has also been reported that long-term use of PPIs paradoxically increases the risk of gastric and colorectal cancer (12-14). Studies show that the use of H2RAs, another antiulcer drug group, may increase the risk of pneumonia, enteric peritonitis, necrotizing enterocolitis, liver cancer, and asthma (15).

Considering the serious side effects of various drugs used for the treatment of PUD in the clinic, the development of drug tolerance, and the recurrent type of this disease, the effectiveness of these drugs has become debatable (16). Therefore, due to the high efficacy and safety of many medicinal plants and active ingredients isolated from these plants, their gastroprotective and antiulcerogenic potential is being investigated and new treatment strategies are being developed (17). 

*Prunus spinosa* L. (Rosaceae)(*P. spinosa*) is a perennial plant that grows in Europe, Asia, and Mediterranean countries and has medicinal uses. It has been frequently used in phytotherapy since ancient times due to its diuretic, antispasmodic, and anti-inflammatory properties (18, 19). In Türkiye, it is used in urinary system diseases, diabetes, liver diseases, and asthma by the public (20, 21). Phenolic compounds (neochlorogenic acid and caffeic acid), flavonoids (quercetin), and anthocyanidins (cyanidin-3-*O*-glucoside, cyanidin 3-*O*-rutoside) were detected in the ethanol extracts of *P. spinosa* fruit. Studies have reported that *P. spinosa* fruits are rich in polyphenolic compounds and vitamin C, thus having a high antioxidant and anti-inflammatory activity (22, 23). In a study, it was shown that *P. spinosa* has antiseptic properties due to the tannins in its composition and shows activity against inflammation of the digestive mucosal layer (24). Another study found that *P. laurocerasus* L. extract belonging to the genus *Prunus* L. had protective effects in the gastric ulcer model induced by indomethacin in rats (25).

 Until now, no *in vivo* anti-ulcer activity studies have been performed on *Prunus* L. spp. Therefore, in this study, we aimed to evaluate the acute protective effect of *P. spinosa* L. ethanolic fruit extract in the indomethacin-induced ulcer model in rats and elucidate the plant’s possible therapeutic mechanisms of action with various biochemical and histopathological analyses. Besides, we aimed to determine the active components responsible for the potential activity of the plant by chromatographic methods.

## Materials and methods

***Supply of plant material***

**Figure 1:**
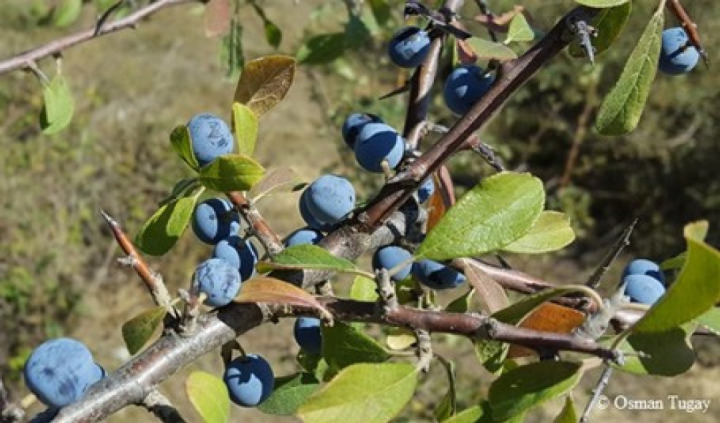
Photo of *Prunus spinosa* L. (Rosaceae)

The fruits of *Prunus spinosa *(Rosaceae) were collected from Seydişehir-Taşagıl around (37°22’56’’ N, 31°51’46’’ E) in the area of Konya in Turkiye in July by Prof Dr Osman TUGAY (O.Tugay 13.561). The plants were identified by Prof Dr Osman TUGAY from the Department of Pharmaceutical Botany, Faculty of Pharmacy, Selçuk University (Konya, Turkiye) and given a Herbarium number (KNYA Herb. No: 30.090)(Figure 1).

***Extraction method***

Fresh fruits of *Prunus spinosa* were cut into small pieces and left to maceration at room temperature by adding 96% ethanol*. *After maceration, the extract was filtered through filter paper. The solvent was then removed to dry under pressure at 40 ^°^C in a rotary evaporator. The obtained extract was stored at +4 ^°^C for later use in biological activity studies and quantitative analysis (26).

***Animal***

Adult male Wistar albino rats (n=60, ~300 grams) used in the study were obtained from Selcuk University Experimental Medicine Research and Application Center (SUDAM). For adaptation to the experimental environment, the rats were kept in quarantine before the experiment. During the entire study, rats were housed in a 12-hour dark/light cycle. Standard feed and water were given *ad libitum*. Special ulcer cages were used to prevent coprophagia. This study was carried out according to ethical rules by considering animal welfare. It was approved by the Animal Experiments Ethics Committee of Selcuk University Experimental Medicine Research and Application Center (Protocol number 2021-46).

***Indomethacin-induced ulcer model***

The ulcer model induced by indomethacin was created by the method described by Kısaoglu *et al*. (27). Briefly, all experimental groups were left to fast 24 hr before drug administration. At the end of the 24th hour of fasting, indomethacin was administered to rats via oral gavage (intragastric, i.g.)(except group-1). The protective efficacy of the *P. spinosa* extracts was compared with the lansoprazole group (group-3) (28). *P. spinosa* extract, the acute protective effect of which was evaluated on the ulcer model induced in rats, was examined in two different doses as low dose (100 mg/kg) and high dose (200 mg/kg)(group-4 and group-5) (29). Lansoprazole, indomethacin and plant extracts were prepared by dissolving them in distilled water.

***Experimental design***

Wistar albino rats were randomly divided into five equal groups:

Group-1 (n=12) Healthy rats: Without the ulcer model being created, only i.g. distilled water was applied

Group-2 (n=12) Indomethacin group: 25 mg/kg i.g. indomethacin was administered 5 min after i.g. distilled water was applied.

Group-3 (n=12) Lansoprazole group: 25 mg/kg i.g. indomethacin was administered 5 min after 30 mg/kg i.g. lansoprazole was applied.

Group-4 (n=12) Low-dose extract group: 25 mg/kg i.g. indomethacin was administered 5 min after 100 mg/kg i.g. *P. spinosa* extract was applied.

Group-5 (n=12) High-dose extract group: 25 mg/kg i.g. indomethacin was administered 5 min after 200 mg/kg i.g. *P. spinosa* extract was applied.

Six hours after the administration of the drugs, the rats were placed under general anesthesia with ketamine and xylazine, and gastric tissues were excised after cervical dislocation. The ulcer areas on the stomach surface were examined macroscopically and measurements were made on millimetric paper. Then, visible lesions were measured to calculate the gastric injury score. The sum of the ulcerous areas was expressed in mm^2 ^as the ulcer score and calculated using the following formula: 

Anti-ulcer effect=% protection=1-[(ulcer score of treatment group / ulcer score of control group)]×100

At the end of the study, all tissues were stored at -80 ^°^C for biochemical analysis and in formaldehyde for histopathological analysis.

***Biochemical analysis***

For biochemical analysis, gastric tissues were stored at -80 ^°^C until the day of analysis. Tissues’ weights were recorded. The tissues were homogenized to 1/10 with PBS (0.01 M, pH=7.4, Sigma catalog no: P4417). Homogenization was performed with Heidolph brand Silent Crusher mechanic homogenizer (Germany). Homogenates were centrifuged at 5000 g for 10 min and obtained supernatants were portioned to analyze concentrations of Tumor Necrosis Factor-α (TNF-α, Bt-Lab cat no: E0764Ra), Interleukin-6 (IL-6, Bt-Lab cat no:E0135Ra), Interleukin-1beta (IL-1β, Bt-lab cat no: E0119Ra), Interleukin-8 (IL-8, Bt-Lab cat no: E1167Ra), Nuclear Factor Kappa B (NF-kB, Bt-Lab cat no: E0287Ra), Prostaglandin E2 (PGE2_, _Bt-Lab cat no: E0504Ra), Vascular Endothelial Growth Factor (VEGF, Bt-Lab cat no: E0659Ra),  Cyclooxygenase 1 (COX-1, Bt-Lab cat no: E1245Ra), Cyclooxygenase 2 (COX-2, Bt-Lab cat no: E0296Ra), and Nitric Oxide (NO, Cayman cat no:10009055)*.* All the analyses were performed following the commercial test kit procedure, with Rayto Microplate Elisa washer (RT-2600; Shenzhen, China) and BMG Labtech (Ortenberg, Germany) Elisa reader. Levels of TNF-α, IL-6, IL-1β, IL-8, NF-kB, PGE2, VEGF, COX-1, and COX-2 were calculated as ng/g tissue and levels of NO were calculated as µM/ g tissue.

***Histopathological analysis***

Gastric tissues obtained from rats were dissected and samples were fixed in 10% buffered formalin for 24 hr. Subsequently, 4 µm sections were cut from paraffin blocks and stained with hematoxylin-eosin. Under an Olympus BX53 light microscope, the following parameters were evaluated and scored: Infiltration of gastric mucosa with neutrophils, mucosal exfoliation, and coagulative necrosis. Depending on the intensity, each parameter was scored as 0: none; 1: mild; 2: moderate; 3: severe

***LC-HRMS assay***

The identification of bioactive substances in *P. spinosa* extract was carried out by using full scan high-resolution accurate mass spectrometry (LC-HRMS). For analyses, the LC system, which contained DIONEX UltiMate 3000 RS pump and autosampler, conducted Exactive Plus Orbitrap (Thermo Fisher Scientific) mass spectrometer equipped with an electrospray ionization (ESI) interface was used. The standard compounds were purchased from Sigma-Aldrich, Merck, and Biosynth in the 95-99% purity range. The stock solutions were prepared by solving compounds in 50% methanol in laboratory conditions. After dilution, compounds at 10 ppb-500 ppb were injected into the LC-HRMS system to create the calibration curve. The extract was then dissolved in 50% methanol and injected into the system. The compounds in the extract were identified by comparing the retention times and exact mass of the standards.

The ions between m/z 60-800 were scanned in the high-resolution mode of the instrument. The standard compounds were in the 95-99% purity range and prepared by solving in 50% methanol. The compounds in the extract were identified by comparing the retention times and exact mass of the standards. 

The following instrument settings were used for analysis: column, Phenomenex® Gemini® 3µm NX-C18 110 Å (100 mm×2 mm); column heat, 30 °C; heat block temperature, 350 ^°^C; DL temperature, 350 ^°^C; nebulizing gas (N2), 7 L/min; collision energy, 25.0. A mixture of 2% acetic acid (A) and methanol (v/v) (B) was selected as the mobile phase. The flow rate was 0.3 ml/min, and the injection volume was 20 µl. The following gradient elution was used, starting at 0% B, then increasing to 98% B in 13.0 min, holding at 98% B for 2.0 min, and then lowering back to 0% B in 16.0 min. The total run time was 20.0 min.

***Statistical analysis***

Statistical analysis was performed using R-4.3.0 and GraphPad Prism 9 programme. Descriptive statistics were presented as minimum-maximum, mean±standart deviation, and median (interquartile deviation). Shapiro-Wilks test was used to test the normality of the data, and the Levene Test was used to test the homogeneity of variances of groups. One-way analysis of variance (One-Way ANOVA) was used to compare means of groups based on normality and homogeneity of variances. The Welch test was used to compare the means of groups based on normality and unequal variances. In addition, the Kruskal-Wallis test was used to compare groups based on non-normality. For multiple comparisons, Tukey and Tamhane Tests were performed. Results of multiple comparisons were shown in Box Plots. Heat map graphs were drawn to display the density of the data distribution according to groups. All analyses were evaluated at α=0.05 significance level (95% confidence level). 

## Results

***Qualitative and quantitative analyses of the bioactive compounds***

**Figure 2:**
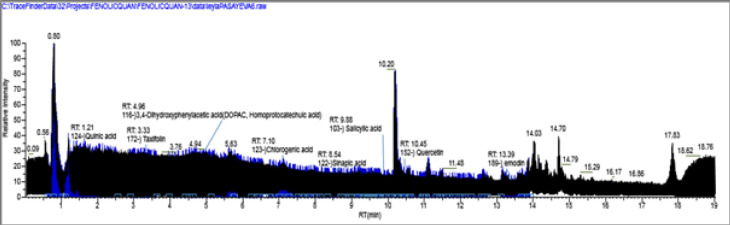
Total ion chromatogram (TIC) profile of *Prunus spinosa* extract and standard compounds

This study studied the bioactive compounds using both positive and negative ionization modes. However, the negative-ion mode provided better sensitivity for these compounds due to more efficient ionization and lower baseline noise. The TIC (Total Ion Chromatogram) of the extract is shown in Figure 2, and the detailed mass parameters of each compound are described in Table 1.

According to the results, among phenolic compounds, ascorbic acid, homoprotocatechuic acid, and genistein were found to be the major compounds in *P. spinosa* extract. The content of the compounds was 1547.521, 1268.217, and 1014.462 µg/g extract, respectively.

***The ulcer area and antiulcer effect of the prunus spinosa extract***

Table 2 shows the effects of *P. spinosa* extract we used in our study at 100 mg/kg (low dose) and 200 mg/kg (high dose) with the data of ulcer area anti-ulcer effect percentage and *P*-values. 

Table 2 shows low and high-dose *P. spinosa* administration has an antiulcer effect compared to the indomethacin group (*P*<0.001). Additionally, lansoprazole has the highest anti-ulcer effect (%99.68). 

***Biochemical results***

**Figure 3:**
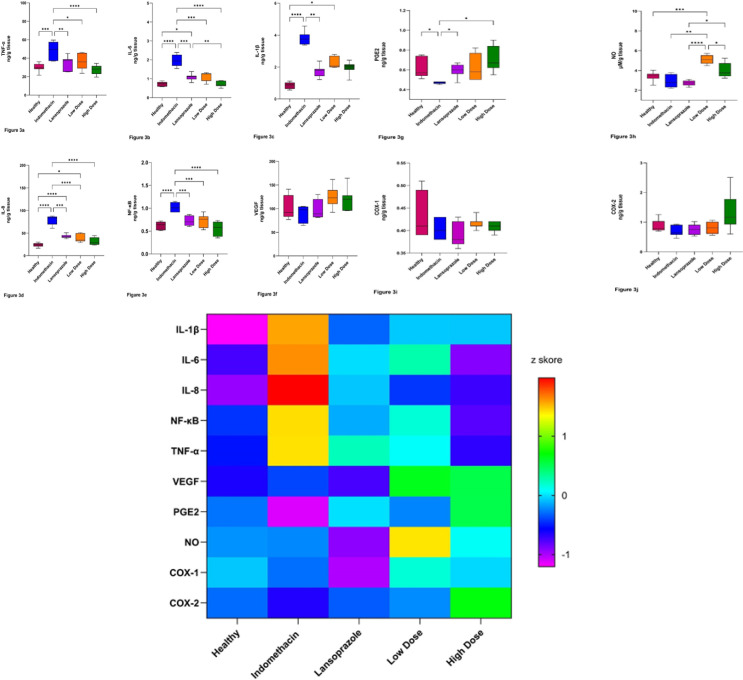
(a-f). a: Comparison of TNF-α levels of study groups in Wistar albino rats, b: Comparison of IL-6 levels of study groups, c: Comparison of IL-1β levels of study groups, d: Comparison of IL-8 levels of study groups, e: Comparison of NF-κB levels of study groups, f: Comparison of VEGF levels of study groups (*P<0.05, **P<0.002, ***P<0.0002, ****P<0.0001), (g-j). Comparison of PGE2 levels of study groups, h: Comparison of NO levels of study groups, i: Comparison of COX-1 levels of study groups, j: Comparison of COX-2 levels of study groups (*P<0.05, **P<0.002, ***P<0.0002, ****P<0.0001), k. Heat map of the changes in the biochemical parameters

As shown in Figures 3a and 3k and Table 3, TNF-α levels (ng/g tissue) were found as 30.03±4.70; 48.58.45±8.98; 34.06±7.72; 35.61±8.39, and 27.23±5.11, respectively, in group 1, indomethacin applied group, lansoprazole applied group, low dose-extract applied group and high dose-extract applied group (mean±SD). It was also determined that the TNF-α levels of Group 1 and Group 2, group 2 and 3, group 2 and 4, and group 2 and 5 notably differed from each other. High-dose extract group levels of TNF-α reached the level of the healthy group. Besides, there were no significant differences between the groups of all doses of extract groups and the lansoprazole group.

According to data of IL-6 (Figures 3b and 3k, Table 3), the highest levels were determined in indomethacin (1.97±0.31 ng/g tissue), whereas the lowest levels (0.72±0.15 ng/g tissue) were seen in high dose extract group. Lansoprazole group levels (1.09±0.18 ng/g tissue) were statistically different (*P*<0.0001) than the levels of the high-dose extract group (0.72±0.15 ng/g tissue) and indomethacin. The healthy group (0.74±0.12 ng/g tissue) and the high-dose extract group have similar values. Thus, the differences were not significantly important. In addition, the low-dose extract group has 1.09±0.25 ng/g tissue levels.

In the evaluation of IL-1β levels, the lowest and highest results were respectively found as 0.85±0.20 and 3.81±0.42 ng /g tissue in the healthy group and indomethacin administered group. The levels of lansoprazole applied group (1.75±0.36 ng/g tissue) were statistically differing from the group of indomethacin (*P*<0.0001). The healthy group’s level was lower than group 2 (*P*<0.0001) and the low-dose extract group (2.25±0.35 ng/g tissue) levels. The value of the high dose extract group was 1.97±0.16 ng/g tissue (Figure 3c, 3k, Table 3) and was found not different from the other groups. 

As shown in Figures 3d and 3k, and Table 3, IL-8 levels were statistically higher in the indomethacin-administrated group (78.48±10.25 ng/g tissue) than in the other groups (*P*<0.0001). There were no significant differences between the lansoprazole applied group (42.75±4.26 ng/g tissue) and both the low-dose extract (40.03±9.58 ng/g tissue) and high-dose extract applied group (31.40±8.38 ng/g tissue). The lowest level of IL-1β was found in the healthy group (23.99±4.40 ng/g tissue), which was significantly different from the other groups except for the high-dose extract applied group (Figure 3d, 3k, Table 3).

NF-kB values (Figures 3e and 3k and Table 3) were found as 0.63±0.084; 1.02±0.09; 0.72±0.10; 0.73±0.14; 0.55±0.16 ng/g tissue, respectively in the healthy group, indomethacin, lansoprazole group, low dose extract applied group, and high dose applied group. The significant differences were determined between indomethacin and lansoprazole, low-dose extract, and high-dose extract applied groups (*P*<0.0001).

The VEGF values did not differ in each group. All the groups’ values were close to each other (*P*>0.05). The levels were 90.88±16.94, 99.77±24.75, 100.78±20.48, 120.08±23.16, and 124.62±22.77 ng/g tissue, respectively, in indomethacin, healthy, lansoprazole, high dose extract, and low dose extract applied group. (Figures 3f and 3k and Table 3).

We found a significant difference between the indomethacin group (0.47±0.01 ng/g tissue) and lansoprazole group (0.60±0.07 ng/g tissue), and the indomethacin and high dose extract applied group (0.71±0.13 ng /g tissue) according to levels of PGE2 (*P*<0.0001). The lowest levels were found in indomethacin. (Figure 3g, 3k, and Table 3).

NO levels were reduced significantly more in the indomethacin (2.93±0.68 µM/g tissue) than in the low dose extract group (5.12±0.45 µM/g tissue, *P*<0.0001) and high dose extract applied group (3.96±0.75 µM/g tissue, *P*<0.0001). There were no significant differences between the healthy group (3.41±0.47 µM/g tissue) and the rest of the groups except the low-dose extract-applied group (*P*<0.0001). The highest levels of NO were found in the low-dose extract applied group. The lansoprazole-applied group showed 2.93±0.68 µM/g tissue levels. There were significant differences between the lansoprazole-applied group and low-dose extract and high-dose extract groups (*P*<0.0001). Moreover, the higher-dose extract applied group has shown lower levels than those of the low-dose extract group (*P*<0.0001)(Figures 3h and 3k, and Table 3).

We determined insignificant differences between all groups according to levels of COX-1 (*P*=0.122) and COX-2 (*P*=0.220). The lowest and the highest levels of COX-1 were respectively found in the lansoprazole group (0,39±0,03 ng/g tissue) and healthy (0,43±0,05 ng/g tissue) group. The levels of COX-1 were respectively 0.40±0.02 ng/g tissue, 0.42 ±0.01 ng/g tissue, and 0.41±0.01 ng/g tissue in indomethacin, low dose extract, and high dose extract applied groups. The lowest and the highest levels of COX-2 were respectively found in the indomethacin group (0.69±0.17 ng/g tissue) and high dose extract applied (1.37±0.64 ng/g tissue) group. COX-2 levels were respectively 0.88±0.20 ng/g tissue; 0.76±0.18 ng/g tissue; 0.80±0.20 ng/g tissue in healthy, lansoprazole and low dose extract applied groups. (Figures 3ı, 3j, and 3k, and Table 3). 

According to the Heat map plot used for each biochemical parameter, color blocks represent Z-score values. The closer the color in the groups to red represents, the higher the Z-score value. Likewise, color similarities indicate that the groups have values close to each other. 

***Histopathological results***

**Figure 4:**
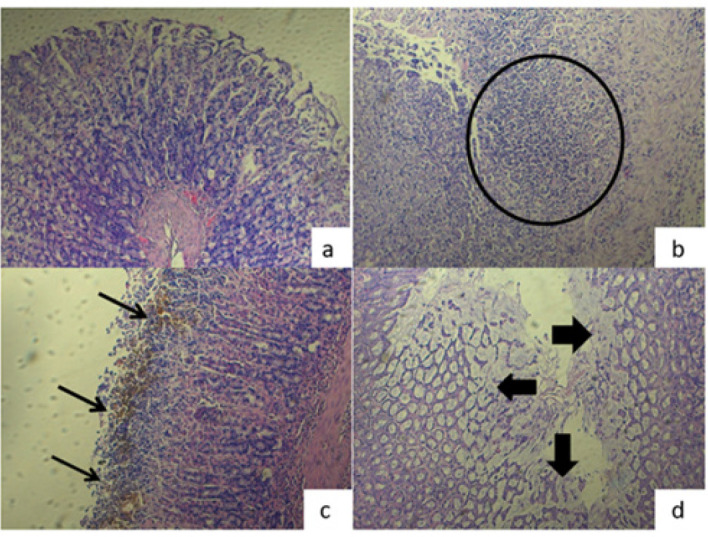
a: Normal gastric mucosa without inflammation (HEX200) in Wistar albino rats, b: Severe neutrophilic infiltration of gastric mucosa in indomethacin group (Circle)(HEX200), c: Mucosal exfoliation and hemosiderin accumulation at mucosa in indomethacin group (Arrows) (HEX200), Fd. Severe exfoliation of gastric mucosa in high-dose extract group (Arrows) (HEX200)

Among histopathologic parameters examined in the present study, a statistically significant difference was revealed between healthy and indomethacin groups regarding neutrophilic infiltration of gastric mucosa. The indomethacin group had increased neutrophilic infiltration of mucosa compared to the control group, which showed acute damage (*P*=0.001)(Figure 4a, b). Comparisons between other groups revealed that the effectiveness of both low- and high-dose *P. spinosa* administration in reducing inflammation was not significant. Nevertheless, the effects of low and high-dose *P. spinosa* administration were the same as those of the lansoprazole and healthy groups. Mucosal exfoliation is another sign of acute damage. Severe exfoliation of the mucosa was observed in indomethacin compared to the healthy group, as expected (*P*=0.013) (Figure 4c). Interestingly, severe mucosal exfoliation was also observed in high dose *P. spinosa *compared to the healthy group, revealing non-effective side of high dose of *P. spinosa* on mucosal damage healing in the acute period (*P*=0.013)(Figure 4d). Besides, the effects of low dose of *P. spinosa* administration was the same as the effects of lansoprazole. In the present study, coagulative necrosis of the gastric mucosa was observed in a few rats mildly. Therefore, no statistically significant difference was found between the groups in terms of necrosis (*P*=0.702)

## Discussion

Although there are many anti-ulcer drugs routinely used in the clinic in the treatment of ulcers, these drugs are insufficient in radical treatment, and the long-term use of the most preferred proton pump inhibitors paradoxically increases the risk of gastric cancer. Therefore, today, the efforts to find alternatives have started with herbal extracts, which have fewer side effects in ulcer treatment. Based on this aspect, in this study, we aimed to investigate whether *P. spinosa* has a protective effect on the stomach in the gastric ulcer model induced by indomethacin in rats. We also tried to enlighten whether there is an effect, which biochemical mechanism minimizes the tissue damage, and how it provides recovery in the acute period by evaluating biochemical and histopathological findings. Therefore, we aimed to elucidate the possible therapeutic action mechanisms of the plant with various biochemical and histopathological analyses. We considered that if the gastric protective activity of the plant extract on gastric ulcers is determined, developing new strategies in the clinic would smoothen the way for the treatment of gastric ulcers. Additionally, up to now, the studies have proven the antioxidant and anti-microbial activity of the extract. Still, no *in vivo* anti-ulcer activity studies have been performed on this species. It is the first literature within the scope of this field so we could not compare our results directly with the other studies.

Some studies have proven that *P. spinosa* has high antioxidant activity due to its polyphenolic compounds (22, 24). It is thought that the activation of the inflammatory process in the stomach and duodenal tissues may be suppressed due to its anti-inflammatory effect (23). Berktas *et al*. have done experimental studies which clarify the antioxidant effects of *P. laurocerasus,* they determined that the lipid peroxidation was high in indomethacin-given rat stomach tissues, also lansoprazole applied group’s levels were the same as those of the healthy group. The researchers indicated that administration of lansoprazole and *P. laurocerasus *fruit extracts showed their protective feature by increasing the antioxidant enzyme activity in the groups (25). When the content of the extract is evaluated in this study; ascorbic acid, the major component in *P. spinosa*, has shown that vitamin C induces less gastric mucosal damage due to the increase in hemoxygenase-1 (HO-1) expression and activity and due to its antioxidant effects. HO-1 plays an important role in gastric protection against NSAID by making cells more resistant to apoptotic death (30, 31). Homoprotocatechic acid, also known as DOPAC, is the other major component in *P. spinosa* fruit extract. Both DOPAC and protocatechuic acid are bioactive components with high antioxidant activity (32). Studies have shown that protocatechic acid has a cytoprotective effect on the stomach and increases mucosal defense (33). Genistein is an isoflavone known for its high antioxidant activity. In anti-ulcer activity studies on genistein, it has been shown to significantly reduce indomethacin-induced ulcers in rats by lowering elevated TNF-α and MPO levels, reducing inflammation and oxidative stress, restoring mucoprotective function, and improving gastric histopathology (34, 35). Considering the anti-ulcer activity studies on the phytochemical compounds such as ascorbic acid (1547.521 µg/g extract), homoprotocatechin (1268.217 µg/g extract) acid, and genistein (1014.462 µg/g extract), which are mainly found in *P. spinosa*, it is predicted that there may be a synergistic effect between the components in terms of ulcer healing activities. In other phytochemical studies conducted on *P. spinosa*, it was found rich in flavonoids and other phenolic compounds such as coumarinic acid, quercetin, apigenin, etc. (19, 36). In another study, vitamin C (between 5.14 and 15.35 mg·100 g^–1^ fw) was detected in *P. spinosa* fruit extract (37). In our study, *P. spinosa* fresh fruit extract was found to be rich in phenolic compounds, similar to previous studies. It is predicted that *P. spinosa* may have high antioxidant and anti-inflammatory effects due to its phenolic components and contribute to ulcer healing. 

The main products are prostaglandin PGE2; for platelets the main product is thromboxane in gastric mucosa. Prostaglandins also protect the microvasculature and can increase the flux of water from serosa to mucosa, via possible dilution of injurious substances. Therefore, it is well known that a number of repair mechanisms, including epithelial cell division and possibly angiogenesis, are prostaglandin dependent. NSAIDs inhibit synthesis of prostanoids by binding to the COX enzyme. Prostaglandin-dependent protective actions that include mucous and bicarbonate secretion, surface epithelial cell hydrophobicity, and mucosal blood flow are inhibited by NSAIDs in the stomach and duodenum. As a consequence of these actions, acute damage and ulcers develop more easily and ulcers heal more slowly when people take NSAIDs (38). While the COX-1 isoenzyme in the arachidonic acid pathway is responsible for the production of mucosa-protective prostaglandin that is responsible for platelet aggregation, and vascular homeostasis (39-42), the COX-2 isoenzyme mainly plays a role in gastric ulcer healing. PGE2, one of the endogenous prostaglandins produced from arachidonic acid, maintains the integrity of the gastric mucosa through its receptors. PGE2 also contributes to the up-regulation of VEGF (39). When we evaluate our findings regarding all above mentioned scientific evidence, it is possible to postulate that our findings support all this knowledge. We determined significantly high levels in lansoprazole group compared to indomethacin group according to level of PGE2. Also, the highest level of PGE2 was in the high-dose extract-applied group (0.71±0.13 ng /g tissue) (*P*<0.0001). The lowest levels were found in the indomethacin group (Figure 3g, Table 3). Therefore, our findings demonstrate that 200 mg/kg *P. spinosa* given to rats normalized the levels to the level of the healthy group. In addition, low-dose extracts applied group’s level reached the healthy group’s levels. Furthermore, *P. spinosa *administration showed potency as the reference drug (lansoprazole) and gave better results than the reference drug. Histopathological results of the mucosal exfoliation have shown that the effects of low and high-dose *P. spinosa* were similar to those of indomethacin administration. Although we cannot directly compare PGE2 with the results of mucosal exfoliation, we support the efficacy of *P. spinosa* via the wound-healing effects of PGE2 in the acute period, especially in the low dose of extract. Pathological results suggest that high doses of *P. spinosa* do not correct the acute damage. Additionally, high-dose extract administration showed acute damage, as in ulcer models, regarding histopathological results; however, the values of PGE2 in high-dose extract administrations were found to be close to those of the healthy group. At this point, we can say that the difference between mucosal exfoliation and biochemical results of PGE2 can be attributed to biochemical changes that are not pathologically reflected in the tissue in an acute state. Besides, various doses of extract having different effects on the damage in the acute state can be expected. As it is known, epithelial cell division and angiogenesis are prostaglandin-dependent. Also, we determined that *P. spinosa *positively affects healing via PGE2 according to biochemical values*. *When we evaluated our findings of COX-1 and COX-2 levels, the lowest and the highest levels of COX-1 were respectively found in the lansoprazole (0.39±0.03 ng/g tissue) and healthy (0.43±0.05 ng/g tissue) groups. Additionally, the lowest and the highest levels of COX-2 were respectively found in the indomethacin group (0.69±0.17 ng/g tissue), and the high dose extract applied (1.37±0.64 ng/g tissue) group (Figures 3i and 3j, and Table 3). We determined insignificant differences between all groups according to levels of COX-1 (*P*=0.122) and COX-2 (*P*=0.220, Table 3). Although the differences between groups were not statistically significant according to COX-1 and COX-2 levels, our data supports the relationship between COX-1 and 2 levels and PGE2. Whereas the levels of COX-1 and COX-2 were higher in the high and low-dose extract administration group, the PGE2 levels were also higher in both dose extract applied groups. From this aspect, our inference is compatible with Takeuchi *et al*. (39). Therefore, it can be said that both doses of extract administration have beneficial effects on ulcer healing, and the extracts have done this healing via the PGE2 pathway. 

When we evaluated the VEGF, we determined that the concentrations did not differ in each group. All of the groups’ values were close to each other (*P*>0.05). The levels were 90.88±16.94, 99.77±24.75, 100.78±20.48, 120.08±23.16, and 124.62±22.77 ng/g tissue, respectively, in indomethacin, healthy, lansoprazole, high dose extract, and low dose extract applied group. (Figures 3f and 3k, and Table 3). Takeuchi *et al*. postulated the contribution of PGE2 to the up-regulation of VEGF (39). It is known that VEGF production begins within 24-72 hr of wound healing (42). According to our results, this point has not been reverberated to the values of VEGF. This may be due to the collection of tissue samples immediately after the ulcer modeling is performed. We think that the results of our study belong to the acute period of effects. So, to figure out the long-term effects of *P. spinosa* on ulcer healing, especially 3-7 days after administration, the extracts may also be investigated for future studies and will provide a clear conclusion about VEGF, which is one of the signs of healing.

It has been shown that TNF-α, NF-κB, and VEGF signaling pathways synergize the gastric mucosa and contribute to the mucosal protective effect. TNF-α and IL-1β up-regulate the expression of COX-2 via the NF-κB pathway (43). In Figure 3a and Table 3, TNF-α levels (ng/g of tissue) were higher in the group treated with indomethacin, while lower levels were observed in the healthy group. The lansoprazole applied group, low dose-extract applied group, and the high dose-extract applied group were shown to have the same levels. High dose extract group levels of TNF-α reached the level of the healthy group. Besides, there were no significant differences between the groups of all doses of extract groups and the lansoprazole group. From this aspect, *P. spinosa *showed anti-inflammatory effects as lansoprazole did in *in vivo* conditions. Sugimoto *et al*. (2010) indicated that the proinflammatory cytokines IL-1β, IL-6, and IL-8 are activated by inflammatory cells in the gastric mucosa, and the analysis of these interleukins is essential in the formation and treatment of gastric ulcers (44). According to our data on IL-6 (Figures 3b and 3k, and Table3), the highest levels were determined in indomethacin, whereas the lowest level was seen in high dose extract group. Lansoprazole group levels were statistically different from the high-dose extract and indomethacin groups. The healthy group (0.74±0.12 ng/g tissue) and the high-dose extract group have similar values, so the differences were not significantly important. In the evaluation of IL-1β levels, the lowest and highest results were respectively found in the healthy group and the indomethacin-administered group. The levels of the lansoprazole-applied group (1.75±0.36 ng/g tissue) differed statistically from the group of indomethacin applied. The healthy group’s level was lower than group 2 and the low dose extract group. The value of the high-dose extract group was 1.97±0.16 ng/g tissue (Figures 3c and 3k, and Table 3) and was found to be not different from the rest of the groups. 

As shown in Figure 3d and Table 3, IL-8 levels were statistically higher in the indomethacin-applied group (78.48±10.25 ng/g tissue) than in the other groups (*P*<0.0001). There were no significant differences between lansoprazole and low-dose extract (40.03±9.58 ng/g tissue) and high-dose extract applied groups (31.40±8.38 ng/g tissue). The lowest level of IL-1β was found in the healthy group (23.99±4.40 ng/g tissue) which was significantly different from the other groups except the high dose extract applied group. (Figures 3d and 3k, and Table 3) Therefore, our data has shown that high dose *P. spinosa* is as effective as lansoprazole with the role of activated anti-inflammatory pathway. All these results belonging to TNF-α, IL-1β, IL-6, and IL-8 demonstrated that *P. spinosa *protected the mucosa via anti-inflammatory effects. Histopathological results of the neutrophilic infiltration of mucosa had an increase in the indomethacin group compared to the control group, which showed acute damage (*P*=0.001) (Figure 4 a, b). Comparisons between other groups revealed that the effectiveness of both low- and high-dose *P. spinosa *administration in reducing inflammation was not significant. However, the effects of low and high-dose *P. spinosa* administration were the same as those of the lansoprazole and healthy groups. We have reached a point where the extract has shown similar effects as lansoprazole in the inflammation pathway, claiming that the data of biochemical and neutrophilic infiltration of mucosa support each other. 

As known, the NF-kB family of transcription factors plays an important role in the expression of several genes implicated in cell growth, inflammation, and apoptosis (40). Cytokines and growth factors are induced by activated NF-κB, and the increasing production and release of inflammatory factors activate NF-κB, therefore aggravating the inflammatory response (45). When we evaluated our results of NF-kB values (Figure 3e, Table 3), we found them as 0.63±0.084, 1.02±0.09, 0.72±0.10, 0.73±0.14, and 0.55±0.16 ng/g tissue, respectively in the healthy group, indomethacin, lansoprazole group, low dose extract applied group, and high dose applied group. The significant differences were determined between indomethacin and lansoprazole, low-dose extract, and high-dose extract applied groups (*P*<0.0001). Likewise, our results are parallel with the results of TNF-α and interleukins. Therefore, these results can be attributed to the effects of P. spinosa as the inflammation inhibitor. 

NO, namely endothelium-derived relaxing factor, is produced because L-arginine and oxygen are converted to L-citrulline under the catalysis of NO synthase (NOS)(45). It is produced by three isoforms of nitric oxide synthase (NOS) enzymes, neuronal NOS (nNOS), inducible NOS (iNOS), and endothelial NOS (eNOS), which are expressed in a cell type and tissue specific manner. NO is produced at high concentrations in response to inflamatory or mitogenic stimuli and reacts with superoxide anions which are highly reactive molecules that induce oxidative damage (46). In accordance with mentioned knowledge above, a study indicated that gastric mucosal NO levels are evaluated as a critical indicator of oxidative damage in the examination of lesions in the gastric mucosa (47). Researchers demonstrated that NO contributes to the synthesis of mucosal protective PGE2 by activating the COX-1 enzyme (48). Szlachcic *et al*. indicated the action of NO as a potent vasodilator known to increase gastric mucosa blood flow; therefore, NO regulates the secretion of mucus and bicarbonate, inhibits the gastric secretion, and protects the gastric mucosa against the damage induced by a variety of damaging agents and corrosive substances in the upper gastrointestinal tract (49). Researchers found that NO from iNOS has a damaging effect on gastric mucosa, while NO from e-NOS has a protective effect against gastric ulcers. Low concentrations of NO have therapeutic effects on gastric ulcers by increasing blood supply to gastric mucosa by dilating local blood vessels (50). Our results belonging to NO levels were significantly lower in the indomethacin group (2.93±0.68 µM/g tissue) than in the low dose extract (5.12±0.45 µM/g tissue, *P*<0.0001) and high dose extract applied groups (3.96±0.75 µM/g tissue, *P*<0.0001). There were no significant differences between the healthy group (3.41±0.47 µM/g tissue) and the rest of the other groups except the low-dose extract-applied group (*P*<0.0001). The highest levels of NO were found in the low-dose extract-applied group. We did not find a significant difference between the lansoprazole-applied group and low-dose extract and high-dose extract groups (*P*<0.0001). Moreover, the higher-dose extract applied group showed lower levels than those of the low-dose extract group (*P*<0.001) (Figures 3h and 3k, and Table 3). When other studies and our findings are evaluated as a whole, the role of NO due to the *P. spinosa *extract and/or dose of extract in our experiment is considered to have short-term effects. As we stated in the Materials and Methods section, we collected the specimen to analyze the gastric tissue six hours after drug administration. When we examine the other studies, the administration was made in time periods expressed in days. Therefore, we cannot compare our data with the other results due to the difference in administration duration of the extract. However, we can say from a biochemical point of view that NO levels are affected by the extract administration duration, and in order to clearly say whether the drug suppresses or increases NO production, we made inferences to create groups in different durations of administration of the extract.

**Table 1:** Compounds in *Prunus* *spinosa* extract identified by LC-HRMS

Compounds	Rt (Min)	[M^−^H]- (*M/Z*)	Content (µg/g_extract_)
Benzoic Acid	8.9	121.02940	15.295
4-Hydroxybenzoic Acid	6.15	137.02442	3.76
Syringic Acid	7.5	121.02975	575.737
Gallic Acid	0.69	169.01425	2.888
Protocatechuic Acid	4.24	153.01933	16.2
Protocatechuic Acid Ethyl Ester	9.11	181.05063	1.336
Homoprotocatechuic Acid (Dopac)	4.96	167.03498	1268.217
Coumaric Acid	8.24	163.04007	20.334
Caffeic Acid	7.12	179.03498	15.986
Chlorogenic Acid	7.1	353.08781	964.554
Quinic Acid	1.21	191.05611	175.685
3-(4-Hydroxyphenyl) Propionic Acid	7.65	165.05572	43.29
L-Ascorbic Acid	0.79	175.02481	1547.521
Rutin	9.19	609.14611	510.11
Luteolin	10.73	285.04046	4.766
Quercetin	10.45	301.03538	82.095
Isoquercitrin (Quercetin 3-Glucoside)	9.24	463.08820	27.557
Narcissin (Narcissoside. Isorhamnetin 3-Rutinoside)	9.78	623.16176	336.165
Isorhamnetin	11.21	315.05103	32.044
Hyperoside	9.24	463.08820	27.557
Astragalin (Kaempferol 3-Glucoside)	9.71	447.09328	136.714
Leucoside (Kaempferol 3-Sambubioside)	9.94	289.06924	624.481
Naringenin	10.47	271.06120	817.946
Eriodictyol	9.32	287.05501	684.113
Liquiritigenin	9.95	255.06628	764.434
Genistein	10.73	269.04555	1014.462
Kuromanine (Cyanidin 3-Glucoside Chloride)	9.71	447.09328	137.773
Esculin Hydrate	6.22	339.07216	7.455
Phloridzin	9.33	435.12967	7.684

**Table 2:** Effects of *Prunus spinosa* and lansoprazole on indomethacin-induced ulcers in Wistar albino rats

Drugs	Dose (mg/kg)	Animals number	Ulcer area (mm^2^)	Antiulcer effect (%)	*p*
*P. spinosa (low dose)*	100 mg/kg	12	15.33±1.75	80.66	<0.001
*P. spinosa (high dose)*	200 mg/kg	12	12.9±1.25	83.73	<0.001
Lansoprazole	30 mg/kg	12	0.25±0.86	99.68	<0.001
Indomethacin	25 mg/kg	12	79.28±2.71	0	-

**Table 3:** Descriptive statistics (min-max, mean±std.dev, median (quartile dev) and *P-values* for biochemical parameters according to groups in Wistar albino rats

Parameters	Groups		Min.-Max.		Mean±std.dev.		Median (quartile dev.)		*P*-value	
IL-1β	Healthy		0.56-1.12		0.85±0.20		0.88(0.20)		*P*<0.0001	
	Indomethacin		3.38-4.57		3.81±0.42		3.75(0.31)			
	Lansoprazol		1.22-2.37		1.75±0.36		1.80(0.22)			
	Low Dose		1.97-2.80		2.25±0.35		2.06(0.36)			
	High Dose		1.18-2.43		1.97±0.16		2.06(0.16)			
IL-6	Healthy		0.58-0.89		0.74±0.12		0.75(0.12)		*P*<0.0001	
	Indomethacin		1.54-2.39		1.97±0.31		1.94(0.30)			
	Lansoprazol		0.80-1.38		1.09±0.18		1.11(0.085)			
	Low Dose		0.71-1.32		1.09±0.25		1.26(0.20)			
	High Dose		0.50-0.90		0.72±0.15		0.67 (0.14)			
NF-κB	Healthy		0.51-0.72		0.63±0.084		0.64( 0.085)		*P*<0.0001	
	Indomethacin		0.91-1.15		1.02±0.09		1.01(0.11)			
	Lansoprazol		0.60-0.86		0.72±0.10		0.70(0.11)			
	Low Dose		0.53-0.92		0.73±0.14		0.76(0.13)			
	High Dose		0.35-0.73		0.55±0.16		0.58(0.16)			
TNF-α	Healthy		21.57-36.18		30.03±4.70		29.80( 2.66)		*P*<0.0001	
	Indomethacin		36.76-59.66		48.58±8.98		49.39(10.28)			
	Lansoprazol		25.24-45.14		34.06±7.72		37.54(6.75)			
	Low Dose		23.76-46.33		35.61±8.39		35.97(8.36)			
	High Dose		19.51-34.35		27.23±5.11		28.22(4.28)			
IL-8	Healthy		16.67-29.95		23.99±4.40		23.99(3.45)		*P*<0.0001	
	Indomethacin		60.63-87.61		78.48±10.25		83.85(8.53)			
	Lansoprazol		37.47-50.76		42.75±4.26		41.87(2.56)			
	Low Dose		29.26-50.99		40.03±9.58		34.50(8.92)			
	High Dose		23.55-44.31		31.40±8.38		28.64(7.96)			
VEGF	Healthy		77.13-141.19		99.77±24.75		91.90(22.64)		0.063	
	Indomethacin		64.54-104.53		90.88±16.94		97.69(17.82)			
	Lansoprazol		81.46-129.67		100.78±20.48		89.09(18.97)			
	Low Dose		92.39-161.91		124.62±22.77		122.82(14.54)			
	High Dose		95.60-164.40		120.08±23.16		120.0(15.88)			
PGE2		Healthy		0.51-0.75		0.63±0.10		0.57(0.10)		*P*<0.0001
		Indomethacin		0.45-0.48		0.47±0.01		0.47(0.01)		
		Lansoprazol		0.47-0.67		0.60±0.07		0.61(0.05)		
		Low Dose		0.50-0.82		0.63±0.14		0.58(0.14)		
		High Dose		0.55-0.90		0.71±0.13		0.67(0.11)		
NO		Healthy		2.52-4.03		3.41±0.47		3.47(0.24)		*P*<0.0001
		Indomethacin		2.20-3.80		2.93±0.68		2.79(0.66)		
		Lansoprazol		2.30-3.11		2.74±0.27		2.75(0.23)		
		Low Dose		4.50-5.72		5.12±0.45		5.09(0.42)		
		High Dose		3.22-5.25		3.96±0.75		3.76(0.65)		
COX-1		Healthy		0.39-0.51		0.43±0.05		0.41(0.06)		0.220
		Indomethacin		0.38-0.43		0.40±0.02		0.40(0.03)		
		Lansoprazol		0.36-0.43		0.39±0.03		0.38(0.03)		
		Low Dose		0.40-0.44		0.42±0.01		0.41(0.01)		
		High Dose		0.39-0.42		0.41±0.01		0.41(0.01)		
COX-2		Healthy		0.70-1.26		0.88±0.20		0.78(0.15)		0.122
		Indomethacin		0.46-0.93		0.69±0.17		0.64(0.16)		
		Lansoprazol		0.53-1.02		0.76±0.18		0.76(0.17)		
		Low Dose		0.56-1.07		0.80±0.20		0.81(0.21)		
		High Dose		0.60-2.51		1.37±0.64		1.17(0.43)		

## Conclusion

This is the first time that an extract of *P. spinosa* was applied to the gastric ulcer in this manner to determine the therapeutical efficiency in gastric ulcer healing. Our results demonstrated that *P. spinosa* protects the gastric mucosa from inflammation and also modulates the PGE2 pathway. When considered in terms of TNF-α, IL-1β, IL-8, IL-6, PGE2, and NF-kB values; it can indicate that it has a similar to or even a more positive effect than the reference substance lansoprazole. Apart from this, we consider that the results of our study belong to the acute period of ulcer healing, and changes in parameters were observed depending on the dose manner. Hence, to figure out the long-term effects of *P. spinosa* on ulcer healing, especially 3–7 days after application, the extracts may also be investigated for future studies and will provide a clear conclusion about VEGF and NO, which are the signs of gastric ulcer healing. 
